# Identification of Risk Factors Associated with Axillary Lymph Node Metastasis for Sentinel Lymph Node-Positive Breast Cancer Patients

**DOI:** 10.1155/2020/8884337

**Published:** 2020-12-29

**Authors:** Zhen He, Xiaowen Lan, Yuting Tan, Xiao Lin, Ge Wen, Xicheng Wang, Xiaobo Huang, Fan Yang

**Affiliations:** ^1^Department of Oncology, The First Affiliation Hospital of Guangdong Pharmaceutical University, Guangzhou 510062, China; ^2^Guangdong Provincial Key Laboratory of Malignant Tumor Epigenetics and Gene Regulation, Medical Research Center, Sun Yat-Sen Memorial Hospital, Sun Yat-Sen University, Guangzhou 510120, China; ^3^Department of Radiation Oncology, Sun Yat-Sen Memorial Hospital, Sun Yat-Sen University, Guangzhou 510120, China; ^4^Department of Breast Tumor Center, Sun Yat-Sen Memorial Hospital, Sun Yat-Sen University, Guangzhou 510120, China; ^5^Department of Radiation Oncology, The Third Affiliated Hospital of Guangzhou Medical University, Guangzhou 510150, China

## Abstract

**Objective:**

This study aimed to identify clinicopathological factors related to the extent of axillary lymph node (ALN) involvement in early-stage BC patients with positive sentinel lymph nodes (SLNs).

**Methods:**

This was a retrospective analysis of 566 patients in cT1-2N0M0 with 1-2 positive SLNs that underwent axillary lymph node dissection (ALND) at Sun Yat-Sen Memorial Hospital. The clinical and pathologic data from these patients were analyzed.

**Results:**

Of these 566 patients, 235 (41.5%) exhibited NSLN metastases. Multivariate analysis revealed that the number of positive SLNs (odds ratio (OR) = 1.511; *P*=0.038), the ratio of metastatic/dissected SLNs (SLN metastasis rate) (OR = 2.124; *P* < 0.001), and lymphovascular invasion (LVI) (OR = 1.503; *P*=0.022) were all independent predictors of NSLN metastasis. Patients with 0, 1, 2, or 3 of these risk factors exhibited NSLN metastases in 29.3%, 35.7%, 50.8%, and 68.3% of cases, respectively. We additionally found that the number of positive SLNs (OR = 3.582; *P* < 0.001), SLN metastasis rate (OR = 2.505; *P*=0.001), LVI (OR = 2.010; *P*=0.004), and HER2 overexpression (OR = 1.774; *P*=0.034) were all independent predictors of N2 disease. When individuals had 0, 1, 2, 3, or 4 of these risk factors, they had four or more involved ALNs in 5.2%, 10.8%, 21.1%, 37.5%, and 70.6% of cases, respectively.

**Conclusion:**

These results suggest that the number of positive SLNs, the SLN metastasis rate, and LVI are all significant predictors of ALN status in BC patients that have 1-2 positive SLNs and that have undergone ALND. In addition, HER2 overexpression was a significant predictor of N2 disease.

## 1. Introduction

Breast cancer (BC) remains one of the most prevalent forms of cancer globally, with 2.1 million people having been diagnosed with this disease in 2018 alone [1]. A number of precision medicine approaches and improved screening efforts have led to significant improvements in the detection of early-stage BC. In patients with early-stage BC, it has thus become important to balance the risks of undertreatment against the potential complications associated with overtreatment [2]. The axillary lymph node (ALN) status of BC patients is the most significant prognostic factor and is a key consideration when determining optimal postoperative radiotherapy and adjuvant systemic treatment strategies in early-stage BC patients. Axillary lymph node dissection (ALND) is a standard approach employed both for tumor staging and for therapeutic purposes and can significantly improve locoregional control in BC patients. Despite these potential benefits, complete ALND is also associated with potential complications including lymphedema, seroma, and numbness. Sentinel lymph node biopsy (SLNB) procedures have markedly altered axilla management strategies in BC patients [3]. When SLNB is negative, complete ALND has been found to not be clinically beneficial [4], whereas complete ALND is routinely performed following positive SLNB. More recent clinical trials have further suggested that ALND may not always be necessary following positive SLNB, as the International Breast Cancer Study Group (IBCSG) 23-01 and the American College of Surgeons Oncology Group (ACOSOG) Z0011 trials revealed that patients with 1-2 involved SLNs that had undergone systemic therapy, adjuvant whole-breast irradiation, and breast-conserving therapies did not benefit from complete ALND [5, 6]. These studies have thus led ALND to not be conducted in women meeting these criteria [7]. There is additional evidence suggesting that only 40% of patients with positive SLNs exhibit additional non-SLN metastasis, with ALND and radiotherapy being unnecessary in the remaining 60% of patients owing to the fact that non-SLN involvement is very rare in early-stage BC [8].

When ALND is not performed, this has the disadvantage of preventing radiation oncologists from being aware of ALN status. The European Organization for Research and Treatment of Cancer (EORTC 10981-22023 AMAROS) trial compared axilla radiotherapy (RT) to ALND in cT1-2N0 patients with limited numbers of positive SLNs and determined that survival outcomes and locoregional control rates between these groups were comparable, with patients that underwent axillary RT treatment exhibiting lower lymphedema incidence [9]. Two concurrent randomized trials evaluating the value of regional nodal irradiation (RNI) in individuals with early-stage breast cancer have found it to be generally beneficial in these patients [10, 11]. However, RNI was also associated with higher rates of lymphedema and other toxicities, and as such it is vital that the potential value of comprehensive nodal RT be carefully balanced against the risk of radiation-associated tissue damage and morbidity. There is therefore potential value in assessing the number of total positive ALNs in BC patients as a means of identifying low- and high-risk patients in order to make more informed regional treatment decisions.

Owing to the above considerations, previous studies have sought to develop a range of predictive models for ALN status in BC patients with positive SLNs [12–15]. However, the utility of these predictive nomograms can vary depending on the specific population being analyzed. The majority of these models also aim to predict whether patients have any positive ALNs (≥1 positive ALNs). There is also clear value in assessing the likelihood of a given patient having N2 disease (≥4 positive ALNs) as a means of guiding treatment planning. This study was therefore designed to identify clinicopathological risk factors associated with the risk of having any positive ALNs and N2 disease in Chinese early-stage BC patients.

The results of this study will be of value to radiation and medical oncologists, as they will support more appropriate treatment planning and radiation fields in early-stage BC patents.

## 2. Materials and Methods

### 2.1. Patients

Clinicopathological data from 566 early-stage BC patients were retrospectively analyzed. All patients had undergone negative clinical ALN palpation, had 1-2 positive SLNs detected upon SLNB, and had undergone subsequent ALND between August 2000 and November 2018 at Sun Yat-Sen Memorial Hospital. Patients were excluded from this study if they were male, had bilateral BC, had inflammatory BC, had a history of prior cancer, or had undergone neoadjuvant chemotherapy.

### 2.2. SLNB

SLNs identification was conducted in all patients using either blue dye or a combination of blue dye and a radiotracer. Stained SLN plotting was conducted using a handheld gamma-detection probe. Nodes that were dyed blue and that had the highest level of gamma activity were identified as SLNs, with positive SLNs being identified via intraoperative frozen section analysis and postoperative hematoxylin and eosin (H&E) staining.

### 2.3. Data Collection

Key clinicopathological parameters from analyzed patients were recorded, including age, primary tumor size, multifocality, histological grade, tumor grade, lymphovascular invasion (LVI), number of positive SLNs, the ratio of metastatic/dissected SLNs (SLN metastasis rate), the number of positive ALNs, operative procedure, estrogen receptor (ER), progesterone receptor (PR), and human epidermal growth factor receptor 2 (HER2) status, Ki-67 index, and immunohistochemistry (IHC)-based subtype. IHC approaches were used to assess ER and PR status in both semiquantitative and quantitative manners [16]. Tumors were considered to exhibit HER2 overexpression when they had IHC scores of 3+ or IHC scores of 2+ that had been confirmed via FISH. Five patient subtypes were identified based on primary tumor IHC findings as follows: Luminal A (ER+, PR+, HER2−, Ki-67 <14%); Luminal/HER2(−) (ER+ or PR+, HER2−, Ki-67 ≥14%); Luminal/HER2(+) (ER+ or PR+, HER2+); HER2 overexpression (ER−, PR−, HER2+); and triple-negative (ER−, PR−, HER2−).

### 2.4. Statistical Analysis

SPSS v19.0 was used for statistical testing. The relationships between clinicopathological factors, NSLN metastasis, and having 4+ axillary metastases were assessed through univariate and multivariate analyses. Chi-squared and Fisher's exact tests were used for univariate analyses, while logistic regression analyses were employed for multivariate analyses. *P* ≤ 0.05 was the significance threshold.

## 3. Results

### 3.1. Clinicopathological Findings

We retrospectively analyzed available clinicopathological data from 566 early-stage BC patients with 1-2 positive SLNs that had undergone ALND. These patients had a median age of 47 years (range: 22–83 years), and an average of 3 SLNs were identified per patient (range: 1–11). More than 6 SLNs were detected in just 6.7% (38/566) of patients, with the remaining patients all having ≤6 SLNs. A total of 420 (74.2%) patients had 1 positive SLN, whereas 146 (25.8%) patients had 2 positive SLNs. A median of 14 NSLNs were retrieved per patient (range: 7–57), with 235 (41.5%) patients exhibiting NSLN metastases and 331 (58.5%) having no evidence of NSLN metastases. The median number of total positive ALNs in this patient population was 2 (range: 1–9), with 1–3 involved ALNs being detected in 465 (82.2%) patients and with 101 (17.8%) patients having ≥4 involved ALNs (Table 1).

### 3.2. Identification of Risk Factors Associated with Any ALN Positivity (≥1 Positive ALN)

In a univariate analysis, we found that the number of positive SLNs (*P* < 0.001), SLN metastasis rate (*P* < 0.001), and LVI (*P*=0.017) were all significantly linked to the likelihood of having any positive ALNs in these BC patients (*P* < 0.05) (Table 1). Subsequent multivariate analysis confirmed that the number of positive SLNs (*P*=0.038; OR = 1.511; 95% CI 1.023–2.232), the SLN metastasis rate (*P* < 0.001; OR = 2.124; 95% CI 1.486–3.036), and LVI (*P*=0.022; OR = 1.503; 95% CI 1.062–2.126) were all independent predictors of NSLN metastasis (Table 2).

**Figure 1:**
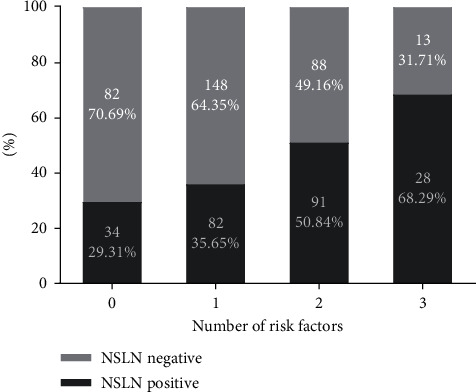
The proportion of patients with the indicated numbers of risk factors exhibiting NSLN positivity (risk factors include LVI, 2 positive SLNs, and SLN metastasis ratio ≥50%). NSLN, nonsentinel lymph node; LVI, lymphovascular invasion; SLN, sentinel lymph node.

The relative rates of NSLN metastasis in patients with 0, 1, 2, or 3 of these risk factors were next determined (Figure 1), with these rates being 29.3%, 35.7%, 50.8%, and 68.3%, respectively.

### 3.3. Identification of Risk Factors Associated with N2 Disease (≥4 Positive ALNs)

In a univariate analysis, the number of positive SLNs (*P* < 0.001), the SLN metastasis rate (*P* < 0.001), LVI (*P*=0.017), HER2 overexpression (*P*=0.006), and IHC-based subtype (*P*=0.05) were all significantly related to N2 disease status in these BC patients (Table 3). Subsequent multivariate logistic regression analysis revealed that the number of positive SLNs (*P* < 0.001; OR = 4.366; 95%CI 2.639–7.223), the SLN metastasis rate (*P*=0.002; OR = 2.432; 95%CI 1.396–4.236), LVI (*P*=0.013; OR = 1.892; 95%CI 1.145–3.127), and HER2 overexpression (*P*=0.037; OR = 1.792; 95%CI 1.036–3.101) were all independent predictors of N2 disease (Table 4).

**Figure 2:**
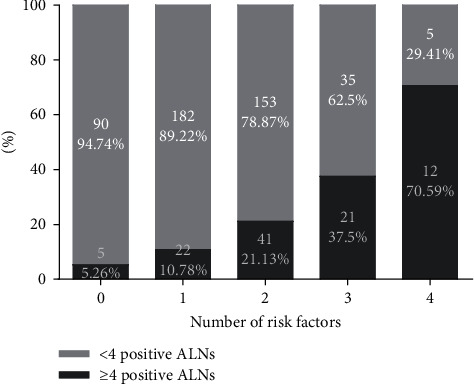
The proportion of patients with the indicated numbers of risk factors exhibiting N2 disease (risk factors include LVI, 2 positive SLNs, SLN metastasis ratio ≥50%, and HER2 overexpression). ALN, axillary lymph node; LVI, lymphovascular invasion; SLN, sentinel lymph node; HER2, human epidermal growth factor receptor 2.

The relative rates of N2 disease in patients with 0, 1, 2, 3, or 4 of these risk factors were next determined (Figure 2), with these rates being 5.2%, 10.8%, 21.1%, 37.5%, and 70.6%, respectively.

## 4. Discussion

In the present study, we sought to identify clinicopathological risk factors associated with the likelihood of having any positive ALNs or N2 disease in early-stage BC patients with 1-2 positive SLNs that had undergone ALND. Our findings indicated that the number of positive SLNs, the SLN metastasis rate, and LVI were independently associated both with having positive ALNs and with N2 disease. Additionally, we found that HER2 overexpression was a significant predictor of N2 disease.

ALN status is a key clinical consideration both for staging purposes and for determining optimal adjuvant treatment regimens. SLNB has become the standard procedure employed for axillary staging in clinically node-negative BC patients. Optimal management strategies in patients with positive SLNs, however, remain controversial. Recent evidence suggests that ALND is not beneficial in presents with 2 or fewer SLN metastases, with the Z0011(5) and IBCSG 23-01(6) trials having detected no differences in survival or locoregional recurrence over a 10-year follow-up period when comparing patients that underwent SLNB only to those that underwent ALND. As such, the American Society of Oncology guidelines recommend that early-stage BC patients with 1-2 positive SLNs not undergo ALND and that they instead be treated via breast-conserving therapy and whole-breast radiotherapy.

Although informative, the patients enrolled in these clinical trials were selected based on rigorous criteria and may therefore not be applicable to the general BC patient population. For example, the median age of patients in the Z0011 trial was 55 years, with 70% of patients having T1 tumors, 83% being ER-positive, 44% having micrometastases, 71% having just one positive SLN, and only 27% of patients in the ALND arm exhibiting additional axillary involvement. Patients in this trial also all underwent breast-conserving therapies, with mastectomy patients not having been included. Similarly, only 9% of patients in the IBCSG 23-01 trial had undergone a mastectomy. ALND remains a common procedure in mastectomy patients when any SLN metastases are identified. Some retrospective analyses have, however, suggested that forgoing ALND in certain mastectomy patients with positive SLNs may be warranted [17, 18]. ALND does not appear to be required in some patients with positive SLNs, and as a consequence, it is essential that individual risk factors be considered in order to strike an appropriate balance between ALN staging and therapy-related complications.

While the Z0011 trial results offer key insights regarding surgical treatments in included patients, they do not offer any conclusive details regarding optimal radiation fields. Standard postoperative radiotherapy was conducted in just 71% of patients in the IBCSG 23-01 trial, with 19% of patients having undergone electron beam-mediated intraoperative radiotherapy without axillary irradiation. In the Z0011 trial, postoperative radiotherapy was conducted in 89% of patients, with half of the patients having undergone high tangential field irradiation and with 18.9% of patients also having undergone prohibited irradiation of the supraclavicular fossa [19]. Coverage of axilla levels I, II, and III by standard tangential fields has been estimated to be 66%, 44%, and 31%, respectively, while these coverage rates are 86%, 71%, and 73% for high tangential fields [20]. The very low axillary progression rates observed in this trial have been associated with the treatment of potential axillary disease via both systemic therapy and high tangential field radiotherapy.

The role of axillary RT as an alternative to ALND was the focus of the OTOASOR study and the AMAROS trial. The multicenter AMAROS trial revealed that axillary RT after positive SLNB provides excellent axillary control for patients with T1-2 primary breast cancer even when ALND is omitted [9]. The smaller, single-center OTOASOR study compared ALND and axillary RT and reached similar results in patients with SLN metastasis. After 8 years of follow-up, there was no statistical difference in axillary recurrence or DFS between the 2 treatment arms [21]. The meta-analysis by Zhao et al. showed that axillary RT is not inferior to ALND in the patients with clinically node-negative breast cancer who had a positive SLN [22]. The AMAROS trials additionally proposed the potential value of a third field, with all patients therein having undergone axillary and infra/supraclavicular irradiation.

Multiple recent trials have also emphasized the value of RNI as an axillary management strategy in BC patients, with both the European Organization for Research and Treatment of Cancer (EORTC) 22922(10) and the National Cancer Institute of Canada MA-20(11) trials having shown that a combination of RNI and whole-breast irradiation can enhance the disease-free survival of early-stage BC patients that had node-positive or high-risk node-negative disease, although this approach did not alter overall survival significantly. Regional radiotherapy was, however, associated with toxicities including lymphedema, and cardiac radiation exposure has been linked to heart damage, making it vital that treatments be carefully planned so as to ensure the heart is not exposed to the radiation field [23]. Given that in many cases radiotherapy offers only modest benefits while causing significant toxicity, it is clear that it should not be administered to all patients that have positive SLNs.

As it is difficult to define optimal radiation fields based on these trials, current NCCN guidelines suggest that positive SLNs who are eligible for Z0011 enrollment may not need to undergo ALND, although further radiotherapy decision-making guidance is limited [24]. Currently, it is recommended that RNI be considered in patients with 1–3 positive ALNs, whereas it is only mandated in those with ≥4 positive ALNs [25]. It is thus important that the number of positive ALNs in BC patients be evaluated in order to guide treatment planning in light of individual risk factors.

Several nomograms have been developed to date with the goal of predicting NSLN metastasis in BC patients exhibiting positive SLNs [12–15]. None of these nomograms, however, have been accepted for clinical utilization owing to their variability with respect to patient demographics and locations. Each clinic must therefore develop its own appropriate nomograms and guidelines for gauging optimal treatment strategies.

Tumor size has generally been found to be a significant predictor of NSLN metastasis [12–15], although this was not the case in our study. The size of SLN metastasis has also been shown to be predictive of NSLN metastasis [12, 14, 15], but we did not detect any such correlation between size of SLN metastasis and ALN metastasis. These inconsistencies in findings may be due to sample size limitations, and many prior studies have also not included tumor size or size of SLN metastasis in their predictive models [26, 27].

There have been countless studies examining the relationship between HER2 status and BC recurrence, with some having shown that HER2 overexpression is closely associated with an elevated risk of ALN metastasis [28, 29]. Consistent with this, we found that HER2 overexpression was closely associated with higher nodal burden, which is in turn a significant predictor of N2 disease.

There is recent evidence indicating that BC molecular subtypes are associated with ALN status [30, 31]. Specifically, triple-negative BC tumors have been shown to be ALN positive more often than other BC types [30]. Wang et al. determined that Luminal B and HER2 overexpressing BC tumors were associated with higher ALN metastasis rates than were Luminal A tumors [31]. However, there have not been sufficient studies exploring the relationship between ALN status and BC molecular subtype in patients with positive SLNs. We did not include sufficient patients with HER2 overexpression subtype BC, and so we were not able to firmly establish whether or not HER2 overexpression subtype is an independent predictor of N2 disease in our multivariate regression analysis.

Our results offer a valuable tool that can be referenced by surgeons and radiation oncologists when they are selecting appropriate treatments for patients with early-stage BC. In patients with 0-1 risk factors that have a low risk of having any positive NSLNs, clinicians may be able to determine that ALND is not warranted and that axillary RT at the I-II level is sufficient to reduce the risk of regional recurrence in light of this risk and other factors including patient age, tumor size, histologic grade, ER status, and PR status [32]. In contrast, in patients with 2-3 risk factors and patients exhibiting HER2 overexpression with a high risk of N2 disease, ALND is more likely to be warranted. Even if ALND is not performed in these patients, our results may allow radiation oncologists to more appropriately select adequate radiation doses and fields. RNI incorporation of a third field including axillary levels I–IV is strongly recommended as a means of minimizing the risk of regional recurrence, given that this approach will ensure better axillary coverage.

This risk of axillary metastatic disease and the degree of nodal involvement must be established as precisely as possible, and modern precision medicine approaches are ideal for evaluating these parameters. A number of genomic assays such as MammaPrint [33], Oncotype DX [34], and EndoPredict [35] have been shown to offer clear prognostic value, aiding in the selection of appropriate adjuvant chemotherapy regimens in early-stage BC patients with ER+ HER2− disease that have 0–3 positive ALNs. While the true utility of these genomic assays in the context of adjuvant RT is still being evaluated [36], they are expected to help with the accurate assessment of locoregional risk in BC patients, thereby guiding optimal radiotherapy regimen planning.

Our results highlight key predictors of residual axillary disease in early-stage BC patients with 1-2 positive SLNs, and as such these findings have the potential to help guide the axillary management of these patients. Even so, this study has multiple limitations. For one, our analyses were retrospective in nature, and they may be limited by a lack of immunohistochemical marker data. Furthermore, all patients in this study were from a single center, and as such further validation of these findings in diverse external patient populations will be required. Future prospective trials will be necessary to evaluate the relationship between nodal burden and both traditional clinicopathological prognostic factors and genomic assays.

In conclusion, herein we demonstrated that the number of positive SLNs, LVI, and SLN metastasis rate were all independent predictors of ALN metastasis in BC patients with 1-2 positive SLNs that had undergone ALND. In addition, HER2 overexpression was a significant predictor of N2 disease. While these results are informative, future nomogram analyses and external validation will be required in order to confirm the relevance of our findings to other clinical populations of BC patients.

## Figures and Tables

**Table 1:** The relationship between clinicopathological characteristics and NSLN metastasis.

Clinicopathological characteristics	Total	NSLN status *n* (%)		Χ^2^	*P*
	(*n* = 566)	Negative	Positive		
	(%)	*n* = 331 (58.5)	*n* = 235 (41.5)		
Age				3.673	0.055
<50	320 (56.5)	176 (55.0)	144 (45.0)		
≥50	246 (43.5)	155 (63.0)	91 (37.0)		

Pathologic tumor size				0.931	0.335
*T*1 ≤2 cm	293 (51.8)	177 (60.4)	116 (39.6)		
*T*2 ≤5 cm	273 (48.2)	154 (56.4)	119 (43.6)		

Tumor type				0.865	0.649
Ductal	530 (93.6)	312 (58.9)	218 (41.1)		
Lobular	11 (1.9)	5 (45.5)	6 (54.5)		
Other	25 (4.4)	14 (56.0)	11 (44.0)		

Nuclear grade				3.286	0.193
I	24 (4.2)	16 (66.7)	8 (33.3)		
II	256 (45.2)	156 (60.9)	100 (39.1)		
III	250 (44.2)	135 (54.0)	115 (46.0)		
N/A	36 (6.4)	24 (66.7)	12 (33.3)		

LVI				4.745	0.029
Present	237 (41.9)	126 (53.2)	111 (46.8)		
Absent	329 (58.1)	205 (62.3)	124 (37.7)		

Multifocality				0.029	0.865
Multifocal/yes	78 (13.8)	47 (60.3)	31 (39.7)		
Unifocal/no	471 (83.2)	279 (59.2)	192 (40.8)		
N/A	17 (3.0)	5 (29.4)	12 (70.6)		

Surgery				0.004	0.953
Conservative	293 (51.8)	171 (58.4)	122 (41.6)		
Mastectomy	273 (48.2)	160 (58.6)	113 (41.4)		

ER receptor status				0.045	0.832
Positive	507 (89.6)	295 (58.2)	212 (41.8)		
Negative	57 (10.1)	34 (59.6)	23 (40.4)		
N/A	2 (0.4)	2 (100)	0 (0)		

PR receptor status				0.119	0.730
Positive	457 (80.7)	265 (58.0)	192 (42.0)		
Negative	107 (18.9)	64 (59.8)	43 (40.2)		
N/A	2 (0.4)	2 (100)	0 (0)		

HER2 expression				0.000	0.999
Positive	117 (20.7)	68 (58.1)	49 (41.9)		
Negative	406 (71.7)	236 (58.1)	170 (41.9)		
N/A	43 (7.6)	27 (62.8)	16 (37.2)		

Ki-67 status				0.813	0.367
<14%	82 (14.5)	53 (64.6)	29 (35.4)		
≥14%	450 (79.5)	267 (59.3)	183 (40.7)		
N/A	34 (6.0)	11 (32.4)	23 (67.6)		

Number of positive SLNs				7.863	0.005
1	420 (74.2)	260 (61.9)	160 (38.1)		
2	146 (25.8)	71 (48.6)	75 (51.4)		

SLN metastasis ratio				19.902	<0.001
<50%	238 (42.0)	165 (69.3)	73 (30.7)		
≥50%	328 (58.0)	166 (50.6)	162 (49.4)		

Size of SLN metastasis				1.378	0.376
Micrometastasis	12 (2.1)	9 (75.0)	3 (25.0)		
Macrometastasis	554 (97.9)	322 (58.1)	232 (41.9)		

IHC-based subtype				2.517	0.642
Luminal A	62 (11.0)	42 (67.7)	20 (32.3)		
Luminal/HER2–	300 (53)	173 (57.7)	127 (42.3)		
Luminal/HER2+	91(16)	53(58.2)	38(41.8)		
HER2(+)	22 (3.9)	12 (54.5)	10 (45.5)		
Triple-negative	19 (3.4)	12 (63.2)	7 (36.8)		
N/A	72 (12.7)	39 (54.2)	33 (45.8)		

NSLN, nonsentinel lymph node; SLN, sentinel lymph node; LVI, lymphovascular invasion; ER, estrogen receptor; PR, progesterone receptor; HER2, human epidermal growth factor receptor 2; IHC, immunohistochemistry.

**Table 2:** The results of multivariate logistic regression analysis of the association between the indicated variables and the likelihood of NSLN metastasis.

	*P* value	OR	95.0% CI	
			Lower	Upper
LVI	0.022	1.503	1.062	2.126
Number of positive SLNs	0.038	1.511	1.023	2.232
SLN metastasis ratio	<0.001	2.124	1.486	3.036

LVI, lymphovascular invasion; SLN, sentinel lymph node; OR, odds ratio; CI, confidence interval.

**Table 3:** The relationship between clinicopathological characteristics and N2 disease.

Clinicopathological characteristics	Total	Axillary status *n* (%)		Χ^2^	*P*
	(*n* = 566)	N1 disease	N2 disease		
	(%)	*n* = 465 (82.2)	*n* = 101 (17.8)		
Age				0.412	0.521
<50	320 (56.5)	260 (81.2)	60 (18.8)		
≥50	246 (43.5)	205 (83.3)	41 (16.7)		

Pathologic tumor size				1.906	0.167
*T*1 ≤2 cm	293 (51.8)	247 (84.3)	46 (15.7)		
*T*2 ≤5 cm	273 (48.2)	218 (79.9)	55 (20.1)		

Tumor type				0.472	0.821
Ductal	530 (93.6)	435 (82.1)	95 (17.9)		
Lobular	11 (1.9)	10 (90.9)	1 (9.1)		
Other	25 (4.4)	20 (80.0)	5 (20.0)		

Nuclear grade				3.251	0.197
I	24 (4.2)	21 (87.5)	3 (12.5)		
II	256 (45.2)	214 (83.6)	42 (16.4)		
III	250 (44.2)	195 (78.0)	55 (22.0)		
N/A	36 (6.4)	35 (97.2)	1 (2.8)		

LVI				5.678	0.017
Present	237 (41.9)	184 (77.6)	53 (22.4)		
Absent	329 (58.1)	281 (85.4)	48 (14.6)		

Multifocality				0.160	0.689
Multifocal/yes	78 (13.8)	64 (82.1)	14 (17.9)		
Unifocal/no	471 (83.2)	395 (83.9)	76 (16.1)		
N/A	17 (3.0)	6 (35.3)	11 (64.7)		

Surgery				1.348	0.246
Conservative	293 (51.8)	246 (84.0)	47 (16.0)		
Mastectomy	273 (48.2)	219 (80.2)	54 (19.8)		

ER receptor status				1.035	0.309
Positive	507 (89.6)	419 (82.6)	88 (17.4)		
Negative	57 (10.1)	44 (77.2)	13 (22.8)		
N/A	2 (0.4)	2 (100)	0 (0)		

PR receptor status				0.055	0.814
Positive	457 (80.7)	376 (82.3)	81 (17.7)		
Negative	107 (18.9)	87 (81.3)	20 (18.7)		
N/A	2 (0.4)	2 (100)	0 (0)		

HER2				7.425	0.006
Positive	117 (20.7)	86 (73.5)	31 (26.5)		
Negative	406 (71.7)	343 (84.5)	63 (15.5)		
N/A	43 (7.6)	36 (83.7)	7 (16.3)		

Ki-67 status				0.687	0.407
<14%	82 (14.5)	71 (86.6)	11 (13.4)		
≥14%	450 (79.5)	373 (82.9)	77 (17.1)		
N/A	34 (6.0)	21 (61.8)	13 (38.2)		

Number of positive SLNs				42.388	<0.001
1	420 (74.2)	371 (88.3)	49 (11.7)		
2	146 (25.8)	94 (64.4)	52 (35.6)		

SLN metastasis ratio				20.723	<0.001
<50%	238 (42.0)	216 (90.8)	22 (9.2)		
≥50%	328 (58.0)	249 (75.9)	79 (24.1)		

Size of SLN metastasis				2.663	0.138
Micrometastasis	12 (2.1)	12 (100.0)	0 (0)		
Macrometastasis	554 (97.9)	453 (81.8)	101 (18.2)		

IHC-based subtype				9.189	0.050
Luminal A	62 (11.0)	54 (87.1)	8 (12.9)		
Luminal/HER2(−)	300 (53)	252 (84)	48 (16)		
Luminal/HER2(+)	91(16)	69 (75.8)	22(24.2)		
HER2(+)	22 (3.9)	14 (63.6)	8 (36.4)		
Triple-negative	19 (3.4)	17 (89.5)	2 (10.5)		
N/A	72 (12.7)	59 (81.9)	13 (18.1)		

SLN, sentinel lymph node; LVI, lymphovascular invasion; ER, estrogen receptor; PR, progesterone receptor; HER2, human epidermal growth factor receptor 2; IHC, immunohistochemistry.

**Table 4:** The results of multivariate logistic regression analysis of the association between the indicated variables and the likelihood of having N2 disease.

	*P* value	OR	95.0% CI	
			Lower	Upper
LVI	0.013	1.892	1.145	3.127
Number of positive SLNs	<0.001	4.366	2.639	7.223
SLN metastasis ratio	0.002	2.432	1.396	4.236
HER2 overexpression	0.037	1.792	1.036	3.101

LVI, lymphovascular invasion; SLN, sentinel lymph node; HER2, human epidermal growth factor receptor 2; OR, odds ratio; CI, confidence interval.

## Data Availability

All data generated or analyzed during this study are included in this published article.
